# NUTRITION, INFLAMMATION, AND PHYSICAL AND MENTAL HEALTH IN AGING ADULTS WITH CHRONIC WOUNDS

**DOI:** 10.1093/geroni/igac059.1620

**Published:** 2022-12-20

**Authors:** Yeji Hwang, Zachary Hathaway, Isabella Stoll, Shelby Hufnal, Peter Lewin, Rose Ann DiMaria-Ghalili

**Affiliations:** Drexel University, Philadelphia, Pennsylvania, United States; Drexel University, Philadelphia, Pennsylvania, United States; Drexel University, Philadelphia, Pennsylvania, United States; Drexel University, Philadelphia, Pennsylvania, United States; Drexel University, Philadelphia, Pennsylvania, United States; Drexel University, College of Nursing and Health Professions, Philadelphia, Pennsylvania, United States

## Abstract

Nutritional status can impact the healing of chronic leg wounds (CLW). This secondary data analysis examined characteristics associated with nutritional status among aging adults (N=62, M= 57.5, SD 12.1 years of age) living with a CLW who were enrolled in a randomized control trial to test a novel ultrasound intervention on wound healing. Nutritional status (Mini-Nutrition Assessment, [MNA]), physical and mental health (36-Item Short-Form Health Status Survey), and inflammation (high-sensitivity C-Reactive Protein [CRP]) were measured at baseline. Majority of the participants were male (54.8%), non-Hispanic Black (67.7%) and either malnourished or at risk of malnutrition (64.5%). Compared to individuals with normal nutrition, individuals with malnutrition or risk for malnutrition had on average poorer physical health (38.7 vs 33.4, p <0.01), mental health (52.4 vs 45.8, p=0.03) and greater inflammation (CRP=13.4 vs 24.0, p=0.05). Tailored interventions targeting nutritional status, inflammation, physical and mental health are needed in aging adults with CLW.

